# Relationship between perioperative thyroid function and acute kidney injury after thyroidectomy

**DOI:** 10.1038/s41598-018-31946-w

**Published:** 2018-09-10

**Authors:** Eun-Young Joo, Yeon Ju Kim, Yonji Go, Jun-Gol Song

**Affiliations:** Department of Anaesthesiology and Pain Medicine, Asan Medical Center, University of Ulsan College of Medicine, Seoul, Republic of Korea

## Abstract

Thyroid dysfunction may alter kidney function via direct renal effects and systemic haemodynamic effects, but information on the effect of thyroid function on postoperative acute kidney injury (AKI) following thyroidectomy remains scarce. We reviewed the medical records of 486 patients who underwent thyroidectomy between January 2010 and December 2014. Thyroid function was evaluated based on the free thyroxine or thyroid stimulating hormone levels. The presence of postoperative AKI was determined using the Kidney Disease: Improving Global Outcomes (KDIGO) criteria. AKI developed in 24 (4.9%) patients after thyroidectomy. There was no association between preoperative thyroid function and postoperative AKI. Patients with postoperative hypothyroidism showed a higher incidence of AKI than patients with normal thyroid function or hyperthyroidism (19.4%, 6.7%, and 0%, respectively; P = 0.044). Multivariable logistic regression analysis showed that male sex (OR, 4.45; 95% CI, 1.80–11.82; P = 0.002), preoperative use of beta-blockers (OR, 4.81; 95% CI, 1.24–16.50; P = 0.016), low preoperative serum albumin levels (OR, 0.29; 95% CI, 0.11–0.76; P = 0.011), and colloid administration (OR, 5.18; 95% CI, 1.42–18.15; P = 0.011) were associated with postoperative AKI. Our results showed that postoperative hypothyroidism might increase the incidence of AKI after thyroidectomy.

## Introduction

Thyroid gland hormones affect almost all organ systems in the body, and their interactions with the kidneys have been well characterized. Thyroid hormones influence renal development and histological changes in kidney structure^1,2^. They also affect physiological functions, including sodium and water homeostasis, renal blood flow, and glomerular filtration rate (GFR)^3,4^. These influences may be mediated via direct renal effects, including the function of many transport systems along the nephron^5–7^, as well as via cardiovascular and systemic haemodynamic effects^8–10^. Thus, both hypothyroidism and hyperthyroidism are associated with marked alterations in kidney function.

Acute kidney injury (AKI) is related to increased health costs and adverse outcomes, including the progression to chronic kidney disease and death^11,12^. Hypothyroidism is associated with elevated serum creatinine levels, as well as reduced GFR and renal blood flow^7,13,14^. Thus, hypothyroidism may induce AKI or contribute to the occurrence of AKI in the presence of other renal insults. Nevertheless, scarce information is available on the relationship between thyroidectomy and postoperative AKI. Hence, we aimed to determine the effect of thyroid function on the prevalence of AKI after thyroidectomy. Moreover, we evaluated factors related to postoperative AKI and the impact of AKI on outcomes following thyroidectomy.

## Results

A total of 486 patients were included in the analysis. Of these patients, AKI was diagnosed in 24 (4.9%) based on the Kidney Disease: Improving Global Outcomes (KDIGO) criteria.

### Patient characteristics and AKI

The preoperative and intraoperative characteristics of these patients are shown in Tables 1 and 2, respectively. There was no association between preoperative thyroid function and the occurrence of postoperative AKI (P = 0.661). Male patients and patients with diabetes mellitus showed a higher incidence of postoperative AKI (P < 0.001 and 0.023, respectively). The preoperative use of beta-blockers was more frequent in patients with postoperative AKI (P = 0.012). Moreover, the postoperative AKI group exhibited longer anaesthesia times (P = 0.019) and more frequent colloid administration. The incidence of AKI after total thyroidectomy was not significantly different than that after hemithyroidectomy (4.7% vs. 6.4%; P = 0.566). AKI incidence was no different between patients who received postoperative T4 replacement and patients who did not (P = 0.286).

**Table 1:** Demographic and preoperative data.

	All patients (n = 486)	Non-AKI group (n = 462)	AKI group (n = 24)	P value
Age (years)	50.3 ± 13.8	50.1 ± 13.9	54.7 ± 11.3	0.116
Sex				<0.001
Male	159 (32.7%)	143 (31.0%)	16 (66.7%)	
Female	327 (67.3%)	319 (69.0%)	8 (33.3%)	
Body mass index (kg/m^2^)	24.5 ± 3.7	24.5 ± 3.7	24.8 ± 3.6	0.681
ASA class				0.139
1	224 (46.1%)	215 (46.5%)	9 (37.5%)	
2	240 (49.4%)	228 (49.4%)	12 (50.0%)	
3	22 (4.5%)	19 (4.1%)	3 (12.5%)	
Hypertension	130 (26.7%)	121 (26.2%)	9 (37.5%)	0.222
Diabetes mellitus	48 (9.9%)	42 (9.1%)	6 (25.0%)	0.023
Cardiovascular disease	16 (3.3%)	16 (3.5%)	0 (0%)	1.000
Medication				
Beta-blockers	30 (6.2%)	25 (5.4%)	5 (20.8%)	0.012
NSAIDs	1 (0.2%)	1 (0.2%)	0 (0%)	1.000
T4	24 (4.9%)	22 (4.8%)	2 (8.3%)	0.335
Haemoglobin (g/dL)	13.3 ± 1.6	13.3 ± 1.6	13.3 ± 1.8	0.988
Creatinine (mg/dL)	0.76 ± 0.21	0.76 ± 0.21	0.82 ± 0.24	0.144
Albumin (g/dL)	4.12 ± 0.38	4.13 ± 0.37	3.88 ± 0.60	0.052
Uric acid (mg/dL)	4.9 ± 1.4	4.8 ± 1.4	5.2 ± 1.2	0.229
Preoperative thyroid function				0.661
Normal	415 (85.4%)	393 (85.1%)	22 (91.7%)	
Hyperthyroidism	30 (6.2%)	29 (6.3%)	1 (4.2%)	
Hypothyroidism	41 (8.4%)	40 (8.7%)	1 (4.2%)	
Tumor size (cm)	1.0 (0.7, 2.0)	1.2 (0.7, 0.9)	1.8 (0.7, 3.2)	0.096
Pathology				0.281
Papillary carcinoma	455 (93.6%)	434 (93.9%)	21 (87.5%)	
Follicular carcinoma	19 (3.9%)	18 (3.9%)	1 (4.2%)	
Medullary carcinoma	7 (1.4%)	6 (1.3%)	1 (4.2%)	
Anaplastic carcinoma	5 (1.0%)	4 (0.9%)	1 (4.2%)	

Data are expressed as mean ± standard deviation, median (interquartile), or number of patients (%), as appropriate.

AKI, acute kidney injury; NSAIDs, non-steroidal anti-inflammatory drugs.

All P values were evaluated by comparing between non-AKI and AKI groups.

**Table 2:** Intraoperative data.

	All patients (n = 486)	Non-AKI group (n = 462)	AKI group (n = 24)	P value
Operation type				0.675
Total thyroidectomy	396 (81.5%)	378 (81.8%)	8 (75.0%)	
Hemithyroidectomy	78 (16.0%)	73 (15.8%)	5 (20.8%)	
Complete thyroidectomy	12 (2.5%)	11 (2.4%)	1 (4.2%)	
Anaesthesia time (min)	255 (160, 329)	255 (160, 322)	347 (193, 495)	0.019
Lowest MBP (mmHg)	67.2 ± 8.5	67.2 ± 8.5	66.1 ± 9.5	0.541
Crystalloid (mL)	700 (500, 1350)	700 (500, 1300)	950 (463, 2588)	0.295
Colloid (%)	50 (10.3%)	42 (9.1%)	8 (33.3%)	0.001
Vasoactive drugs	78 (16.0%)	71 (15.4%)	7 (29.2%)	0.086

Data are expressed as mean ± standard deviation, median (interquartile), or number of patients (%), as appropriate.

AKI, acute kidney injury; MBP; mean blood pressure, RBC, red blood cell.

All P values were evaluated by comparing between non-AKI and AKI groups.

### Thyroid function and AKI

There was no association between preoperative thyroid function and the occurrence of postoperative AKI (P = 0.661). For 72 patients, thyroid function was evaluated within 7 postoperative days. There was a higher incidence of AKI among patients with postoperative hypothyroidism than among patients with normal thyroid function or hyperthyroidism (19.4%, 6.7%, and 0%, respectively; P = 0.044).

### Risk factors for AKI

Multivariable logistic regression analysis indicated that male sex (odds ratio [OR], 4.45; 95% confidence interval [CI], 1.80–11.82; P = 0.002), preoperative use of beta-blockers (OR, 4.81; CI, 1.24–16.50; P = 0.016), low preoperative serum albumin levels (OR, 0.29; 95% CI, 0.11–0.76; P = 0.011), and colloid administration (OR, 5.18; 95% CI, 1.42–18.15; P = 0.011) were associated with the occurrence of postoperative AKI (Table 3).

**Table 3:** Univariate and multivariable regression analyses to identify factors associated with acute kidney injury after thyroidectomy.

Variables	Univariate analysis		Multivariate analysis	
	Odds ratio (95% CI)	P value	Odds ratio (95% CI)	P value
Male	4.46 (1.92–11.23)	<0.001	4.45 (1.80–11.82)	0.002
Diabetes mellitus	3.33 (1.16–8.44)	0.016		
Beta-blockers	4.60 (1.43–12.57)	0.005	4.81 (1.24–16.50)	0.016
Albumin	0.26 (0.11–0.64)	0.002	0.29 (0.11–0.76)	0.011
Tumour size	1.23 (0.98–1.49)	0.046		
Pathology	1.70 (0.82–2.97)	0.091		
Crystalloid	1.00 (1.00–1.00)	0.025		
Colloid	5.00 (1.93–12.09)	<0.001	5.18 (1.42–18.15)	0.011
Vasoactive drugs	2.27 (0.85–5.46)	0.080		

Odds ratios and 95% confidence intervals (CI) are expressed.

The variables with P < 0.1 in univariate analyses were entered into the multivariable logistic regression model.

### Outcomes

Based on outcome analyses, patients with AKI were more likely to stay longer in hospital (6.5 [4,11] days vs. 5 [4,7] days; P = 0.040) than patients without AKI (Table 4). None of the patients with the postoperative AKI needed renal replacement therapy.

**Table 4:** Postoperative outcomes.

	All patients (n = 486)	Non-AKI group (n = 462)	AKI group (n = 24)	P value
Intensive care unit admission	37 (7.6%)	33 (7.1%)	4 (16.7%)	0.100
Hospital stay (days)	5 (4, 7)	5 (4, 7)	6.5 (4, 11)	0.040

Data are expressed as median (interquartile), or number of patients (%), as appropriate.

AKI, acute kidney injury.

All P values were evaluated by comparing between non-AKI and AKI groups.

## Discussion

In this study cohort, 4.9% of the patients undergoing thyroidectomy developed AKI based on the KDIGO criteria. There was a higher incidence of AKI in patients with postoperative hypothyroidism than in patients with normal thyroid function or hyperthyroidism. Multivariable analysis indicated that male sex, preoperative use of beta-blockers, low serum albumin levels, and colloid administration were associated with the occurrence of AKI. Moreover, postoperative AKI was associated with longer hospital stays.

The occurrence of postoperative AKI raises major concerns regarding patient safety, but few studies to date have assessed AKI incidence after thyroidectomy. Previous studies have reported that AKI develops in 0.8–10% of patients after non-cardiac surgeries^15–17^. Additionally, Recent studies have also shown that AKI develop in 4.4% of patients after unilateral total knee arthroplasty^18^. After colorectal surgery, AKI has also been shown to develop in 9.6% of patients based on the Acute Kidney Injury Network (AKIN) criteria, and in 5.5% of patients based on the Risk, Injury, Failure, Loss, and End-stage Renal Failure (RIFLE) criteria^19^. The prevalence of AKI in the present study is consistent with previous reports, although it should be noted that thyroidectomy is a relatively low-risk surgery in terms of bleeding or haemodynamic instability.

The impact of thyroid dysfunction on renal function has been emphasized in recent studies. Thyroid hormones play important roles in renal development and the function of many transport systems along the nephron^1,2,5,6^. They also affect water and electrolyte metabolism, as well as cardiovascular function^3,4,9^. All these effects lead to important alterations in renal function in both hyperthyroidism and hypothyroidism. Serum creatinine levels are lower in cases of hyperthyroidism, whereas contrast findings are noted in cases of hypothyroidism. The renal impairment associated with hypothyroidism is primarily believed to be a result of reduced cardiac output and the subsequent decrease in the renal blood flow and GFR^7,13,14,20,21^. Thus, hypothyroidism may contribute to the exacerbation of pre-existing chronic kidney disease or the occurrence of AKI in the presence of other renal insults. Before radioiodine scanning for thyroid cancer follow-up, patients must stop taking levothyroxine and placed in a hypothyroid state. Kreisman *et al*. reported that such patients show a consistent elevation of serum creatinine levels in the hypothyroid state, and that this elevation is reversible after replacement of levothyroxine^7^. Our study showed comparable results in that patients with postoperative hypothyroidism exhibited a higher incidence of AKI than patients with normal thyroid function or hyperthyroidism (19.4%, 6.7%, and 0%, respectively).

The time over which AKI develops in patients with hypothyroidism remains unknown. In a previous study, the serum creatinine levels were found to be elevated within 2 weeks of the onset hypothyroidism^7^. We defined AKI using the KDIGO criteria based on the serum creatinine level within 7 days, and this may be an insufficient period to detect the effect of postoperative hypothyroidism on postoperative AKI. Nevertheless, the association between postoperative thyroid function and serum creatinine level should be carefully considered by clinicians, because hypothyroidism can lead to AKI in patients with normal preoperative creatinine levels. Although the elevation of serum creatinine levels typically normalizes following thyroid hormone replacement after a short period of hypothyroidism, slower and incomplete recovery has been noted in cases with more prolonged periods of severe hypothyroidism^20^. Furthermore, the changes in renal function in the hypothyroid state may also lead to potential alterations in therapeutic drug doses^7^.

Our multivariable analysis agreed with previous studies in terms of factors associated with AKI, including male sex, preoperative use of beta-blockers, low serum albumin level, and colloid administration. Albumin is known to have a renoprotective effect, mediated by antioxidant and anti-inflammatory properties^22,23^. Moreover, it functions as a reservoir for signalling molecules and donors of nitric oxide (NO) that enhance the renal blood flow and GFR by dilating vessels and improving renal function^24^. Furthermore, albumin tends to improve the microcirculatory performance that supports the maintenance of major organ functions^25^. Thus, both preoperative and postoperative hypoalbuminaemia are major risk factors for AKI in many previous studies^18,26–28^. Although controversial, beta-blockers have similar effects to albumin on renal function. Despite the concerns about haemodynamic effects including a decrease in renal blood flow, several beta-blockers are known to mitigate renal injury by antioxidant properties or activating NO synthase. Several animal studies have reported that beta-blockers reduce the severity of AKI or have renal protective effects^29–31^. However, Le Manach *et al*. reported that the use of preoperative beta-blockers was associated with an increased frequency of renal failure because beta-blockers limit the increase of compensated cardiac output when major blood loss occurs^32^. In a number of studies of the effects of beta-blockers on advanced liver diseases, patients receiving beta-blockers had a high probability of developing AKI, and this was related to the inhibited cardiac compensatory reserve^33,34^. These harmful effects of beta-blockers could be related to renal hypoperfusion^35^. Furthermore, beta-blockers are recommended as third-line antihypertensive agents in patients with proteinuria according to the Kidney Disease Outcomes Quality Initiative (K/DOQI) guidelines^36^. Thus, the effectiveness of beta-blockers against postoperative AKI among patients in normal haemodynamic states after minor surgery remains unclear. Further studies are required to clarify the effects of beta-blockers on renal function.

The detrimental effect of colloid administration on renal function remains a major concern. The oncotic force of these solutions may decrease renal filtration pressure, and this may inhibit renal function^37^. Another potential pathologic mechanism involves renal interstitial proliferation, macrophage infiltration, and tubular damage contributing to hydroxyethyl starch-induced nephrotoxicity^38^. Previous studies have shown the adverse renal effects of colloid administration in critically ill and septic patients^39,40^, although sufficient evidence on this topic is not available for healthy patients under perioperative care. A retrospective study of 174 patients who underwent orthotropic liver transplantation showed a higher incidence of AKI after colloid administration as compared to albumin administration^41^. In contrast, another study showed no association between intraoperative colloid administration and increased AKI risk after living donor hepatectomy^42^. Despite the relatively healthy patient characteristics in the present study, colloid administration was associated with AKI after thyroidectomy. However, additional studies with a randomized, controlled design are needed to clarify the findings regarding the nephrotoxicity of colloid administration in surgical patients.

The prevalence of underlying diabetes mellitus was higher in the AKI group, but it showed no statistical relationship with AKI following multivariable analysis. Diabetes is also known as one of the risk factors of for postoperative AKI; and this association is thought to result from the possibility of pre-existing CKD^43^. This may affect our results because we excluded patients with CKD. Additionally, the development of postoperative AKI is related to the type of operation. Previous reports about the relationship between diabetes and postoperative CKD showed inconsistent results varying by procedure^44^. Lastly, the low incidence of postoperative AKI in our study may have affected the multivariable analysis findings.

The retrospective observational study design resulted in some important limitations. As serum creatinine was not measured on every single day of postoperative admission and follow-up, there might be some undetected cases of postoperative AKI. Nevertheless, the incidence of postoperative AKI in this study was 4.9%, which is consistent with previous reports. Additionally, in accordance with KDIGO guidelines, the frequency of serum creatinine and urine output measurements to detect AKI should be individualized based on patient risk^45^. A lack of urine analysis, including sodium concentration and proteinuria, can be another concern. The analysis of urine sodium concentration can identify the cause of AKI after thyroidectomy. Although the renal impairment associated with hypothyroidism is primarily believed to be a result of reduced cardiac output and the subsequent decrease in renal blood flow and GFR, thyroid hormone is also known to affect kidney function by direct effects on the renal tubular system. Additionally, the presence of proteinuria in diabetes can result in the loss of thyroid hormone, and diabetes itself can contribute to AKI incidence. Although we considered as many variables as possible and performed multivariable analysis to obtain reliable results, we could not eliminate the possibility of residual confounding variables. Additionally, the low incidence of postoperative AKI among patients involved in this retrospective study limited the power to detect the relationship between thyroid function and AKI, as well as the effects of the investigated variables on AKI. Nevertheless, this was a suitable strategy for evaluating the effect of thyroid function on postoperative AKI in the absence of prospective studies. Further prospective studies with well-constructed designs for clarifying the effect of thyroid function on postoperative AKI are needed.

In conclusion, AKI developed in 4.9% of patients who underwent thyroidectomy. We found that there was a higher incidence of AKI among patients with postoperative hypothyroidism than among patients with normal thyroid function or hyperthyroidism after thyroidectomy. As the knowledge of the association between postoperative thyroid function and postoperative AKI may have important clinical implications, further prospective studies should be conducted to clarify the effect of thyroid function on postoperative AKI incidence in thyroidectomy patients.

## Methods

**Figure 1:**
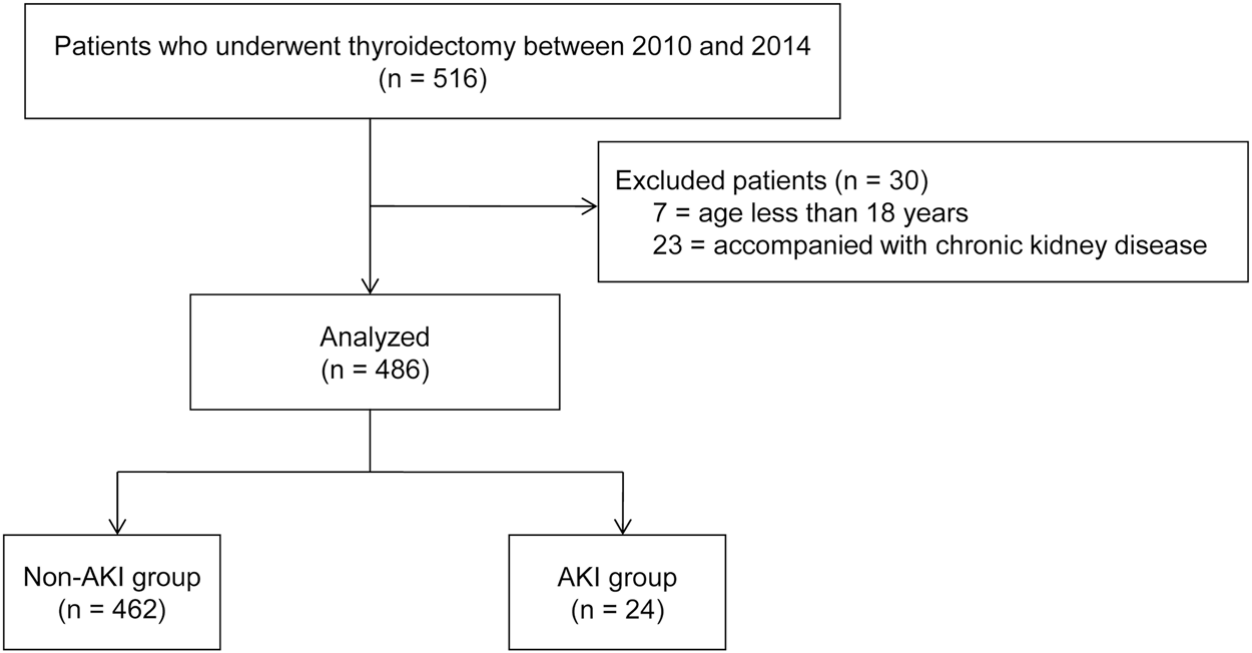
Study flow diagram. AKI, acute kidney injury.

After approval was obtained from the Institutional Review Board of Asan Medical Center, we reviewed the records of all patients who underwent thyroidectomy for thyroid cancer at Asan Medical Center, Seoul, Republic of Korea, between January 2010 and December 2014. Informed consent was waived due to the retrospective nature of our study. Of the 516 identified patients, we excluded those aged <18 years (n = 7) and those with chronic kidney disease (n = 23). Thus, a total of 486 patients were finally included in the present study (Fig. 1). This manuscript adheres to the STROBE guidelines.

We collected information regarding the baseline characteristics and laboratory, intraoperative, and postoperative data from the computerized patient record system at our institution (Asan Medical Center Information System Electronic Medical Records). Baseline characteristics included sex, age, body mass index, comorbidities (hypertension, diabetes mellitus, and cardiovascular disease), and the use of prescribed medications (beta-blockers and levothyroxine). Pathological diagnosis, tumour stage, and tumour size were also included as cancer characteristics. Laboratory data included sodium, potassium, chloride, calcium, haemoglobin, albumin, uric acid, and serum creatinine levels. To evaluate thyroid function, free thyroxine (FT4), and thyroid stimulating hormone (TSH) levels were recorded. Levels of serum TSH and FT4 were measured using the TSH-CTK-3 immunoradiometric assay (IRMA) kit (DiaSorin S.p.A, Saluggia, Italy) and fT4 radioimmunoassay (RIA) kit (Beckman Coulter/Immunotech, Prague, Czech Republic), respectively. Hyperthyroidism was defined as having a TSH level < 0.45 mIU/L with normal FT4 levels or FT4 levels >2.0 ng/dL. Hypothyroidism was defined as having a TSH level >4.5 mIU/L with normal FT4 levels or FT4 levels <0.8 ng/dL^46^. The type of thyroidectomy and lymph node dissection performed was also recorded. Recorded intraoperative data included anaesthesia time, lowest mean blood pressure, volume of administered fluids, and use of vasoactive drugs. Anaesthesia time was defined as the time from anaesthesia induction to the transfer of the patient from the operating room. Intraoperatively, additional fluid or vasoactive drug administration were considered if systolic blood pressure was maintained below 80 mmHg.

The primary outcome of this study was the prevalence of AKI based on the Kidney Disease: Improving Global Outcomes (KDIGO) criteria. According to the KDIGO criteria, AKI was defined as an increase in the serum creatinine level by ≥0.3 mg/dL within 48 hours or an increase in serum creatinine by ≥1.5 times within 7 days^45^. Serum creatinine was measured on days 1, 2, 3, 5, and 7 after surgery and at least one time during that period in all patients. We did not use the urinary output criterion due to the unreliability of urine output measurements. The other outcome variables included the occurrence of postoperative intensive care unit (ICU) admission and the duration of hospital stay.

### Statistical analysis

Data are presented as mean ± standard deviation, median (interquartile range), or number (percentage), as appropriate. The χ^2^-test or Fisher’s exact test was used to compare categorical variables in postoperative AKI groups. Continuous variables in these two groups were compared using the t-test, or the Mann-Whitney *U* test if the distribution was not normal. To identify the risk factors for postoperative AKI, logistic regression analysis was used to calculate ORs with 95% CIs. All variables in Tables 1 and 2 were tested, and variables with P < 0.1 after univariate analysis were entered into the multivariable logistic regression model. The final models were determined by backward elimination procedures with P < 0.05 as model retention criteria. All P values less than 0.05 were considered statistically significant. All statistical analyses were performed using SPSS Statistics (version 21; IBM Corp, Chicago, IL).

## Data Availability

All data generated or analysed during this study are available from the corresponding author upon reasonable request.
